# *CYP2D7* Sequence Variation Interferes with TaqMan CYP2D6^*^15 and ^*^35 Genotyping

**DOI:** 10.3389/fphar.2015.00312

**Published:** 2016-01-12

**Authors:** Amanda K. Riffel, Mehdi Dehghani, Toinette Hartshorne, Kristen C. Floyd, J. Steven Leeder, Kevin P. Rosenblatt, Andrea Gaedigk

**Affiliations:** ^1^Division of Clinical Pharmacology, Toxicology and Therapeutic Innovation, Children's Mercy Kansas CityKansas City, MO, USA; ^2^CompanionDx® Reference LabHouston, TX, USA; ^3^Division of Oncology, Department of Internal Medicine, University of Texas Health Science Center at HoustonHouston, TX, USA; ^4^Genetic Analysis, Genetic Sciences Division, Thermo Fisher ScientificSouth San Francisco, CA, USA; ^5^School of Medicine, University of Missouri-Kansas CityKansas City, MO, USA

**Keywords:** CYP2D6, CYP2D7, TaqMan, genotyping, CYP2D6^*^15, CYP2D6^*^35, CYP2D6^*^43

## Abstract

TaqMan™ genotyping assays are widely used to genotype *CYP2D6*, which encodes a major drug metabolizing enzyme. Assay design for *CYP2D6* can be challenging owing to the presence of two pseudogenes, *CYP2D7* and *CYP2D8*, structural and copy number variation and numerous single nucleotide polymorphisms (SNPs) some of which reflect the wild-type sequence of the *CYP2D7* pseudogene. The aim of this study was to identify the mechanism causing false-positive CYP2D6^*^15 calls and remediate those by redesigning and validating alternative TaqMan genotype assays. Among 13,866 DNA samples genotyped by the CompanionDx® lab on the OpenArray platform, 70 samples were identified as heterozygotes for 137Tins, the key SNP of CYP2D6^*^15. However, only 15 samples were confirmed when tested with the Luminex xTAG CYP2D6 Kit and sequencing of *CYP2D6*-specific long range (XL)-PCR products. Genotype and gene resequencing of *CYP2D6* and *CYP2D7*-specific XL-PCR products revealed a CC>GT dinucleotide SNP in exon 1 of *CYP2D7* that reverts the sequence to *CYP2D6* and allows a TaqMan assay PCR primer to bind. Because *CYP2D7* also carries a Tins, a false-positive mutation signal is generated. This *CYP2D7* SNP was also responsible for generating false-positive signals for rs769258 (CYP2D6^*^35) which is also located in exon 1. Although alternative CYP2D6^*^15 and ^*^35 assays resolved the issue, we discovered a novel CYP2D6^*^15 subvariant in one sample that carries additional SNPs preventing detection with the alternate assay. The frequency of CYP2D6^*^15 was 0.1% in this ethnically diverse U.S. population sample. In addition, we also discovered linkage between the *CYP2D7* CC>GT dinucleotide SNP and the 77G>A (rs28371696) SNP of CYP2D6^*^43. The frequency of this tentatively functional allele was 0.2%. Taken together, these findings emphasize that regardless of how careful genotyping assays are designed and evaluated before being commercially marketed, rare or unknown SNPs underneath primer and/or probe regions can impact the performance of PCR-based genotype assays, including TaqMan. Regardless of the test platform used, it is prudent to confirm rare allele calls by an independent method.

## Introduction

TaqMan genotyping assays are widely used within research and clinical testing laboratories. Due to the availability of pre-designed assays, genotyping using this technology is relatively straight forward, allows customization of individual assays into panels (such as the OpenArray platform, Thermo Fisher Scientific, Waltham, MA) and is automatable (Mein et al., 2000; Ranade et al., 2001; Johnson et al., 2012; Fedick et al., 2013). Owing to these advantages, TaqMan technology is widely employed for *CYP2D6* genotype analysis. Among all drug metabolizing enzymes, *CYP2D6* is probably the most structurally and genotypically complex, and extensively studied gene (Zanger et al., 2004, 2008; Ingelman-Sundberg, 2005; Ingelman-Sundberg et al., 2007; Ingelman-Sundberg and Sim, 2010; Teh and Bertilsson, 2012; Zanger and Schwab, 2013). It metabolizes numerous clinically-used drugs, including many antidepressants, antipsychotics, and opioids (Zhou, 2009a,b). Pre-emptive *CYP2D6* genotype testing is increasingly used to guide therapy (Dunnenberger et al., 2015), or used to explain adverse drug reactions or treatment failure after treatment initiation (Zhou et al., 2015).

Assay design for *CYP2D6*, however, is arduous due to the presence of two pseudogenes, *CYP2D7* and *CYP2D8*, within the gene locus, structural and copy number variation and the presence of numerous single nucleotide polymorphisms and insertion/deletion variations (collectively referred here as SNPs; Gaedigk, 2013). Hence, it is challenging to identify genome-unique regions in the vicinity of target SNPs to design robust TaqMan genotyping assays. SNPs located underneath a primer or TaqMan probe binding site can affect an assay's performance. As recently reported, SNPs within the amplicon can also impact assay performance (Gaedigk et al., 2015a).

Although most test panels include only the SNPs defining the most common *CYP2D6* alleles, others routinely test for rare or extremely rare alleles, including CYP2D6^*^15 (http://www.ncbi.nlm.nih.gov/gtr/). This allele is characterized by a nucleotide insertion in exon 1 (137Tins) that causes a frameshift and leads to premature translation termination. Of note, per definition the *CYP2D7* gene also carries two Ts at the homologous position, which is a hallmark feature rendering this pseudogene nonfunctional. CYP2D6^*^15 was first described in a German population (Sachse et al., 1996), but it has not been detected in other European populations. Although data for this allele are sparse, it has been reported in Brazilians of European ancestry (Kohlrausch et al., 2009), Lengua Native Americans of Paraguay (Bailliet et al., 2007) and a Mexican population (Alcazar-González et al., 2013), but not in other world populations. Based on the available literature, this nonfunctional allele has an extremely low frequency of < 0.5%. A summary of allele frequencies can be found in Clinical Pharmacogenetics Implementation Consortium (CPIC) guidelines (Hicks et al., 2013, 2015; Crews et al., 2014) or via the web at http://www.pharmgkb.org/download.action?filename=CYP2D6_Frequency_Table_and_Legend_R3.pdf. Due to the rarity of certain alleles, it is therefore difficult to get hold of gDNA reference material for assay development and quality control. Consequently, it is important to verify assay results to avoid reporting false-positive results that may cause wrongful genotype assignments and may lead to inaccurate predictions of a subject's phenotype status.

In contrast to CYP2D6^*^15, the CYP2D6^*^35 allele is more commonly observed. The latter can be identified by a SNP that is also located in exon 1 and represents a “key” allelic feature for identification (31G>A, rs769258, V11M). This SNP was first reported in 1997 (Marez et al., 1997) and is most commonly noted in Europeans with an average frequency of 6%. Its frequency is much lower or absent in other populations. CYP2D6^*^35 is often not tested for because it is deemed fully functional. In the absence of testing for this variant, a CYP2D6^*^1 or ^*^2 will be assigned by “default” depending on test design.

TaqMan assays were extensively validated by CompanionDx® using Coriell DNA and hundreds of DNA samples which were confirmed by orthogonal methods such as the Luminex xTAG CYP2D6 platform and Sanger sequencing. No false-negative calls were detected during these validation efforts. Subsequently, however, we identified samples testing positive for the CYP2D6^*^15 allele (137Tins) in a large cohort of subjects residing in the U.S. Verification of the results with alternative methods uncovered false-positive CYP2D6^*^15 calls.

The aim of this study was to identify the factors responsible for the false-positive genotype calls and to remediate those by redesigning and validating alternative TaqMan genotype assays.

## Materials and methods

### Subjects and samples

DNA was isolated from buccal swab samples of a large, ethnically diverse U.S. population of 13,866 consecutive subjects (*n* = 27, 740 alleles). Samples from 20 subjects who gave written informed consent and had positive TaqMan CYP2D6^*^15 calls, were selected for follow-up studies (Table 1). Due to limited amounts of gDNA, Whole Genome Amplification (WGA) was performed using 15 ng of gDNA and the GenomiPhi DNA Amplification kit (GE Healthcare Life Sciences, Pittsburgh, PA USA) as recommended by the supplier. WGA-DNA was diluted 1:25-fold for subsequent testing of TaqMan genotype assay performance.

**Table 1:** Summary of CYP2D6^*^15 genotyping and sequencing results with the initial and alternate TaqMan genotyping assays.

**Sample#**	**Ethnicity**	Initial CYP2D6^*^15 assay^a^			**Genotype**		***Sequencing***	**Sequencing**	Alternate CYP2D6^*^15 assay^a^		
		**OpenArray^b^**	***CYP2D6* XL-PCR**	***CYP2D7* XL-PCR**	**OpenArray^h^**	**Luminex**	***CYP2D7* XL-PCR**	***CYP2D6* XL-PCR**	**gDNA**	***CYP2D6* XL-PCR**	***CYP2D7* XL-PCR**
1	Unknown	^*^15 het	^*^15 het	No signal	^*^2A/^*^15	No call	CC	^*^15 het	^*^15 het	^*^15 het	No signal
2	African American	^*^15 het	wt	^*^15 mut	*1/^*^17	^*^1/^*^17	CC/GT	wt	wt	wt	No signal
3	Unknown	^*^15 het	wt	^*^15 mut	*1/^*^2B	^*^1/^*^2	CC/GT	wt	wt	wt	No signal
4	Caucasian	^*^15 het	wt	^*^15 mut	*1/^*^4, dup^c^	^*^1/^*^4, dup^c^	CC/GT	wt	wt	wt	No signal
5	Hispanic	^*^15 het	wt	^*^15 mut	^*^1/*1	^*^1/^*^1	CC/GT	wt	No gDNA	wt	No signal
6	Unknown	^*^15 het	wt	^*^15 mut	*1/^*^2D^h^	^*^1/^*^29	GT	wt	wt	wt	No signal
7*^f^*	Caucasian	^*^15 het	wt	no result	^*^1/^*^2, dup^c^	^*^1/^*^2, dup^c^	no result	wt	no result	no result	No result
8	African American	^*^15 het	wt	^*^15 mut	*1/^*^17	^*^1/^*^17	CC/GT	wt	wt	wt	No signal
9	Hispanic	^*^15 het	wt	^*^15 mut	*1/^*^2, dup^c^	^*^1/^*^2, dup^c^	CC/GT	wt	No gDNA	wt	No signal
10	African American	^*^15 het	wt	^*^15 mut	*1/^*^17	^*^1/^*^17	CC/GT	wt	No gDNA	wt	No Signal
11	Caucasian	^*^15 het	^*^15 het	No signal	^*^2A/^*^15	^*^2/^*^15	*CC^e^*	^*^15 het	No gDNA	^*^15 het	No Signal
12	African American	^*^15 het	wt	^*^15 mut	*1/^*^17, dup^c^	No call	CC/GT*^e^*	wt	No gDNA	wt	No signal
13^g^	Caucasian	^*^15 het	^*^15 het^d^	No signal	^*^4/^*^15	^*^4/^*^15	*CC^e^*	^*^15 het^d^	wt	wt^d^	No signal
14	Caucasian	^*^15 het	^*^15 het	No signal	^*^15/^*^41	^*^15/^*^41	*CC*	^*^15 het	^*^15 het	^*^15 het	No signal
15	African American	^*^15 het	wt	^*^15 mut	^*^2A/^*^17	^*^2/^*^17	CC/GT*^e^*	wt	wt	wt	No signal
16	Hispanic	^*^15 het	wt	^*^15 mut	^*^1/^*^17	^*^1/^*^17	CC/GT	wt	wt	wt	No signal
17	African American	^*^15 het	wt	^*^15 mut	^*^1/*1	^*^1/^*^1	CC/GT	wt	No gDNA	wt	No signal
18	Caucasian	^*^15 het	wt	^*^15 mut	^*^1/*1	^*^1/^*^1	CC/GT*^e^*	wt	No gDNA	wt	No signal
19	African American	^*^15 het	wt	^*^15 mut	*1/^*^41	^*^1/^*^41	CC/GT*^e^*	wt	No gDNA	wt	No signal
20	African American	^*^15 het	wt	^*^15 mut	*1/^*^17	^*^1/^*^17	CC/GT	wt	No gDNA	wt	No signal
		**TaqMan genotyping**			**TaqMan**						
NA17128^i^		^*^15 het	wt	^*^15 mut	^*^1/*1	n/d	CC/GT*^e^*	wt	wt	wt	No signal
PGx-A^i^		wt	wt	No signal	^*^2/^*^41	n/d	CC	wt	wt	wt	No signal
PGx-B^i^		wt	wt	No signal	^*^4/^*^6	n/d	CC	wt	wt	wt	No signal

a *initial and alternate assay IDs are C__32407245_40 and C__32407245_60, respectively*.

b OpenArray genotyping results were obtained with the original CYP2D6^*^15 TaqMan assay.

c *Sample was positive for a duplication event, but platform does determine on which allele the duplication is located*.

d *The result with the alternate TaqMan genotyping assay is discordant*.

e *CYP2D7 variation identified by sequencing and confirmed by HRM*.

f *Sample did not yielded results for all follow-up studies*.

g Novel CYP2D6^*^15 variant.

h CYP2D6^*^29 could not be assigned with the TaqMan assay set used in this analysis. All samples with a red “*1” were false-positive for ^*^15 and also positive for 77G>A suggesting the presence of a CYP2D6^*^43 (see Suppl Table 1).

i *Genotyped using TaqMan genotyping assays in 96-well format. het, heterozygous; mut, homozygous for variant; no signal, no fluorescent signal was detected indicating that no amplicon has been generated; n/d, not determined*.

*Note that the CYP2D7 CC>GT variation is inventoried by dbSNP under rs386821512 and annotated as GG>CA on the forward genome strand*.

NA17128 was obtained from the Coriell Institute for Medical Research (Camden, NJ). Two de-identified DNA samples from a repository maintained at Children's Mercy (PGx-A and B) served as controls. The use of repository samples was approved by the Institutional Review Board of Children's Mercy Kansas City.

### TaqMan genotyping

Initial genotype analysis of the 13,866 samples was performed with commercially available TaqMan assays using the OpenArray platform on a QuantStudio 12K Flex instrument (Thermo Fisher Scientific; formerly Life Technologies, Grand Island, NY). The OpenArray included assays testing of the following SNPs: −1584C>G (rs1080985), 100C>T (rs1065852), 124G>A (rs5030862), 137Tins (rs72549357), 883G>C (rs201377835), 1023C>T (rs28371706), 1707T>del (rs5030655), 1758G>T/A (rs5030865), 1846G>A (rs3892097), 2549delA (rs35742686), 2613_2615delAGA (rs5030656), 2850C>T (rs1694700, 2935A>C (rs5030867), 2988G>A (rs28371725), and 4180G>C (rs1135840). CYP2D6^*^15 (137Tins) was detected using TaqMan assay ID C__32407245_40. Follow-up TaqMan genotyping for CYP2D6^*^15 (137Tins; assay ID C__32407245_40) and ^*^35 (31G >A, rs769258; assay ID C_27102444_80) was performed on a QuantStudio 12K Flex instrument using 96-well plates and the TaqMan Genotyping Mastermix Mix (Thermo Fisher Scientific-Life Technologies, Grand Island, NY) as recommended by the manufacturer.

Follow up studies were also conducted with alternative assays for CYP2D6^*^15 and CYP2D6^*^35 (to be commercialized with assay IDs C__32407245_60 and C_27102444_F0, respectively). The PCR primers in these redesigned assays were moved to alternate binding sites flanking each mutation, which contained nucleotide differences between *CYP2D6* and *CYP2D7* sequences to drive *CYP2D6*-specific amplification and avoid nonspecific amplification of *CYP2D7* caused by primer overlap with the *CYP2D7* rs386821512 SNP in the original assays. Assay performance was tested on gDNA, WGA-DNA and *CYP2D6* and *CYP2D7* XL-PCR templates using the 96-well format as described below. In addition, a custom TaqMan SNP genotyping assay to detect 77G>A, which is part of the CYP2D6^*^43 and ^*^46 haplotypes, was run on *CYP2D6* XL-PCR templates.

Long-range (XL)-PCR was performed to specifically amplify the *CYP2D6* and *CYP2D7* genes in their entirety. Briefly, *CYP2D6*-specific, 6.6 or 6.7 kb long fragments were generated with primers pairs forward 5′ GGCTCCAAGCATGGCAGCTGC and reverse 5′CGACTGAGCCCTGGGAGGTAGGTAG and forward 5′ TCACCCCCAGCGGACTTATCAACC and reverse 5′CGACTGAGCCCTGGGAGGTAGGTAG, respectively, essentially as previously described; this fragment is referred to as fragment A in previous reports (Gaedigk et al., 2007, 2015b). The 6.6 kb-long fragment was used for sequence-based confirmation of CYP2D6^*^15 TaqMan assay results; the 6.7 kb long fragment A, which amplifies more robustly, was generated for all other follow-up studies and evaluation of the alternate TaqMan genotyping assays. The 4.4 kb long fragment for *CYP2D7* was generated with primers 5′ TCCGACCAGGCCTTTCTACCAC and 5′ CACCAGAAAGCTGACGACACGAGA. An 8 μl reaction contained 15 ng gDNA, 5% DMSO, 500 nM of each primer, and a 1X final concentration of Kapa Long Range HotStart Master Mix (Kapa Biosystems, Wilmington, MA). PCR cycling parameters included an initial denaturation for 3 min at 94°C, followed by 35 cycles of denaturation at 94°C for 20 s, annealing at 68°C for 30 s and extension at 68°C for 7 min. To assure *CYP2D6* or *CYP2D7* amplicon generation, 2 μl aliquots were analyzed by agarose gel electrophoresis. PCR fragments were diluted 2000-fold with 10 mM Tris pH 7 to serve as templates in TaqMan and High Resolution Melt (HRM) assays.

### Luminex genotyping

To confirm CYP2D6^*^15-positive results, genotyping was conducted on the FDA-cleared Luminex platform with the xTAG CYP2D6 Kit V3 (Luminex Corporation, Austin, TX) following the provided protocol. Briefly, the assay incorporates multiple PCR reactions and allele-specific primer extension assays. All assays were read on the Luminex LX200 device using IS 3.1.971 software and the data were analyzed by xTAG CYP2D6 V3 data analysis software version 1.22. Additional assay details including the alleles tested can be found at the company's website. This platform was chosen because it utilizes a different SNP detection technology than TaqMan.

### DNA sequencing

For confirmation, a 6.6 kb long fragment A was generated from 66 of the 70 samples that were found positive for CYP2D6^*^15 and partially sequenced over the exon 1 region harboring the 137Tins. *CYP2D6* exon 1 was amplified using the predesigned primer pairs forward 5′ATGTATAAATGCCCTTCTCCAGGAA and reverse 5′GCAGGTTCACTCACAGCAGAG (Hs00225801_CE, Thermo Fisher Scientific). PCR products were sequenced using the BigDye Direct Cycle Sequencing Kit (Life Technologies) and a 3730xl DNA Analyzer instrument (Life Technologies).

The exon 1 region of a *CYP2D7*-specific XL-PCR product from selected samples was also sequenced with BigDye chemistry on a 3730xl DNA Analyzer. The *CYP2D6* and *CYP2D7* genes of NA17128 were sequenced in their entirety.

DNA sequences were aligned to M33388 and AY545216 (reference sequences for *CYP2D6*) and to four unique *CYP2D7* sequences: M33387, ENS00000205702 (corresponding to ENS00000612115 and NC_000022.11 of GRCh38.p2), ENS00000263181 and ENS00000278088. There is no recognized reference sequence for *CYP2D7* at this time; ENS00000205702 was set as a reference because this sequence represents *CYP2D7* in GRCh38.

### High resolution melt (HRM) analysis

To facilitate screening of *CYP2D7* rs386821512 (CC>GT; positions −1 and −2 in respect to the ATG start codon; GRCh38 42144465:42144466) a HRM assay was developed. Briefly, a 164 bp amplicon was generated with primers forward 5′ GTCACGCGCTCGGTGTGCTGAG and reverse 5′ CCACCAGGAGCAGGAAGATTGCCAC. Six μl reactions contained 400 nM of each primer, 2.5 mM MgCl_2,_ and 0.6 μl of 1:2000-fold diluted XL-PCR amplicon. The reaction was cycled at an initial denaturation of 95°C for 3 min, followed by 30 cycles of denaturation at 95°C for 5 s, annealing at 72°C for 10 s and extension at 72°C for 20 s. Melt curve analysis was performed using the Eco Real-Time PCR System (Illumina, San Diego, CA). Note that the primers used for this assay are not *CYP2D7*-specific and, therefore, this assay must be performed on a *CYP2D7* XL-PCR template and not gDNA. NA17128 served as positive control.

### *CYP2D6* nomenclature

Allele designations throughout the manuscript are according to those defined by the Human Cytochrome P450 (*CYP*) Allele Nomenclature Database (Sim and Ingelman-Sundberg, 2013) at http://www.cypalleles.ki.se/.

## Results

Among a large, diverse U.S. population of 13,866 samples, 70 samples (0.5%) initially tested positive for CYP2D6^*^15 when genotyped on the OpenArray platform (Suppl Table 1). Of those, 19 self-identified as Caucasian, 34 as African American, 7 as Hispanic, 1 as Asian and 9 were of unknown ethnicity.

To confirm the presence of this relatively rare nonfunctional allele, all 70 samples which returned positive results for CYP2D6^*^15 were genotyped with the Luminex xTAG system. For 66 samples, the *CYP2D6*-specific 6.6 kb long XL-PCR product was generated and the exon 1 region sequenced; the remaining samples failed to produce the amplicon. Fifteen samples were confirmed CYP2D6^*^15-positive; among those were 11 Caucasians, one Asian and three of unknown ethnicity. Fifty-one samples were negative for CYP2D6^*^15 by sequencing and Luminex assay (Table 1 and Suppl Table 1).

**Figure 1:**
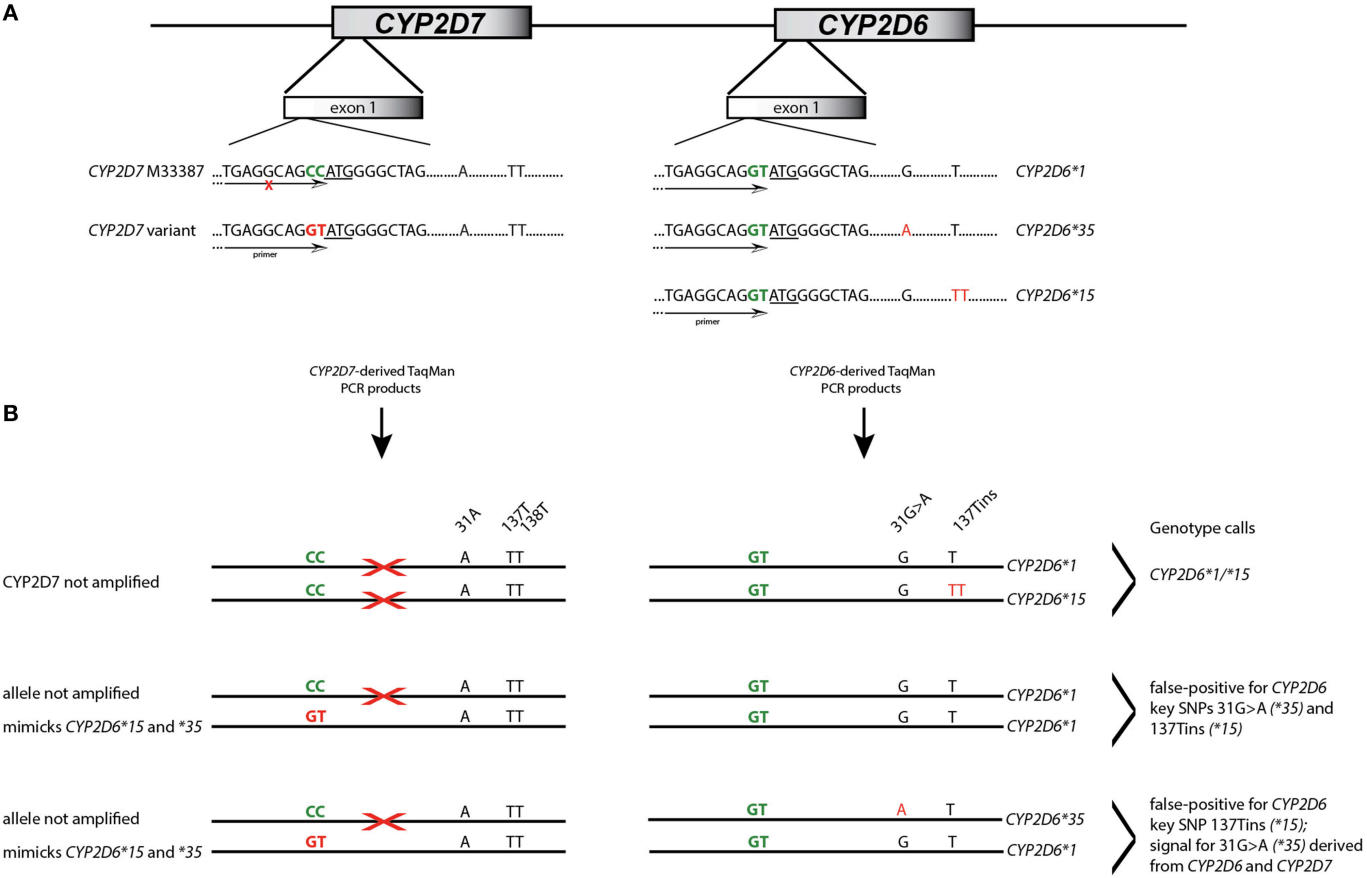
Interference of *CYP2D7* variation on CYP2D6^*^15 and ^*^35 TaqMan genotype assay results. **(A)** Shows the exon 1 region of *CYP2D6* and the *CYP2D7* pseudogene. Sequence differences at the −1 and −2 positions are highlighted in green and the ATG translation start codon is underlined. *CYP2D7* carries a CC at positions −1 and −2 while *CYP2D6* has GT. This difference was employed for primer design for the initial CYP2D6^*^15 TaqMan genotyping assay. SNPs defining allelic variants are shown in red; nucleotide positions are as indicated. The primer location is provided by the arrow. An “X” indicates that the primer does not generate PCR product. **(B)** Details the mechanism by which false-positive assay results are generated. The first example shows a correct CYP2D6^*^15 call; the TaqMan primer only binds to *CYP2D6*. In the second example, one *CYP2D7* allele carries GT allowing primer binding and PCR product amplification. Since the *CYP2D7* derived amplicon has the T-insertion and 31A, the assay yields a false-positive results for CYP2D6^*^15 and ^*^35. In the third example, CYP2D6^*^15 will also be false-positive. Regarding 31G>A, fluorescent signals will be generated from the variant *CYP2D7* allele as well as the CYP2D6^*^35 allele which may lead to a shift in cluster position.

**Figure 2:**
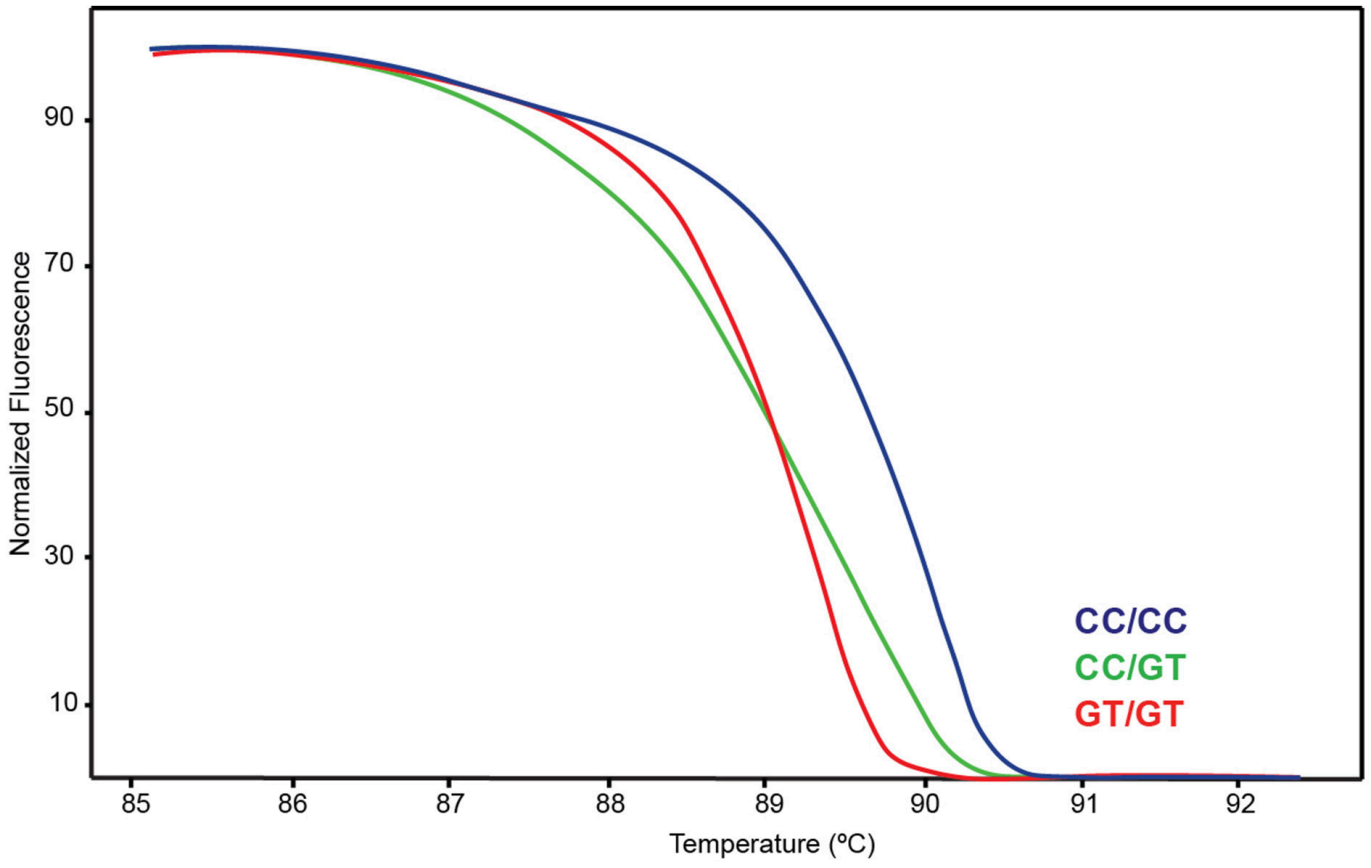
**High Resolution Melt (HRM) assay detecting variation on *CYP2D7*-specific XL-PCR template**. Blue indicates wt, green heterozygous and red variant.

A Coriell sample (NA17128) was independently observed by one of the authors to be heterozygous for CYP2D6^*^15 when genotyped with TaqMan from gDNA. These observations led us to hypothesize that nonspecific amplification from the *CYP2D7* pseudogene may cause these miscalls. To explore this, 20 of the 70 samples and NA17128 were selected for further analysis along with two samples, PGx-A and PGx-B, that were previously genotyped as negative for CYP2D6^*^15 and ^*^35 (Table 1). Sequence analysis of the *CYP2D7* region of interest revealed CC>GT SNPs in exon 1 of *CYP2D7* at positions −1 and −2 relative to the ATG start codon in false-positives and NA17128 (Figure 1A). This sequence variation was also present in all other false-positive samples as subsequently determined by HRM analysis that was performed on a XL-PCR template encompassing the *CYP2D7* gene (Figure 2). As illustrated in Figure 1B, the *CYP2D7* CC>GT SNPs revert the sequence to *CYP2D6* and thereby allowed one of the CYP2D6^*^15 TaqMan genotyping assay PCR primers to bind and support the generation of an amplicon from *CYP2D7*. Because *CYP2D7* also carries an additional T in exon 1 (the *CYP2D6* 137Tins in fact corresponds to the *CYP2D7* reference sequence), a mutation signal is generated, triggering the false-positive CYP2D6^*^15 calls.

Interestingly, NA17128 was also heterozygous for CYP2D6^*^35 (31G>A), but lacked other SNPs such as −1584C>G (rs1080985), 2850C>T (rs16947), or 4180G>C (rs1135840) known to be linked with 31G>A (Gaedigk et al., 2003). We therefore suspected that the *CYP2D7* CC>GT variation may also interfere with the CYP2D6^*^35 genotyping TaqMan assay, resulting in false-positive calls as well given that the CYP2D6^*^15 and ^*^35 assays share a primer binding site that overlies the dinucleotide change. Indeed, all samples with a false-positive CYP2D6^*^15 call presented as false-positive for CYP2D6^*^35 when genotyped on gDNA or WGA-DNA and were negative when genotyping was performed on *CYP2D6*-specific XL-PCR amplicons suggesting that the *CYP2D7* CC>GT dinucleotide SNP is indeed the underlying cause for the false-positive results.

These conclusions were further corroborated by running both TaqMan genotyping assays on *CYP2D7*-specific template. As expected, those carrying CC>GT produced a “homozygous” signal (only the variant *CYP2D7* allele amplified, mimicking homozygosity) while those not having CC>GT did not produce a fluorescence signal.

**Figure 3:**
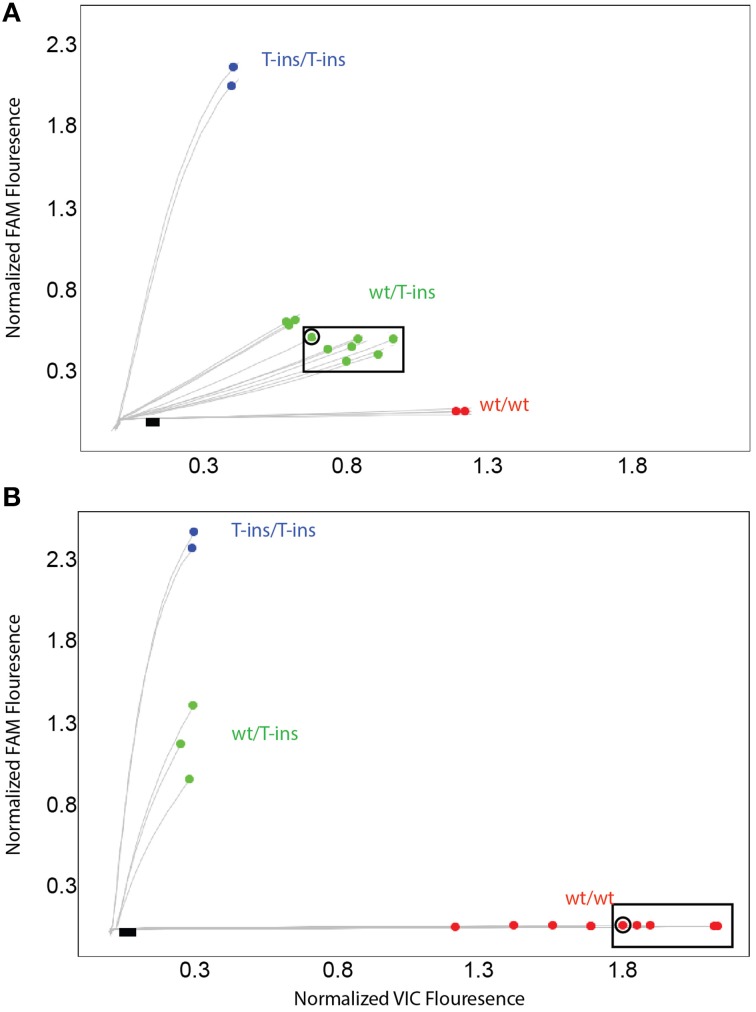
TaqMan scatter plots generated with the initial (A) and alternate (B) CYP2D6^*^15 TaqMan genotyping assay for false-positive and true positive samples. Profiles shown were generated on WGA-DNA. Boxed samples are false-positives with the initial assay **(A)** that have been resolved with the alternate TaqMan genotyping assay. **(B)** Black dot in symbol indicates sample 13 carrying the novel CYP2D6^*^15var. This subject was false-negative with the alternate CYP2D6^*^15 genotyping assay.

**Figure 4:**
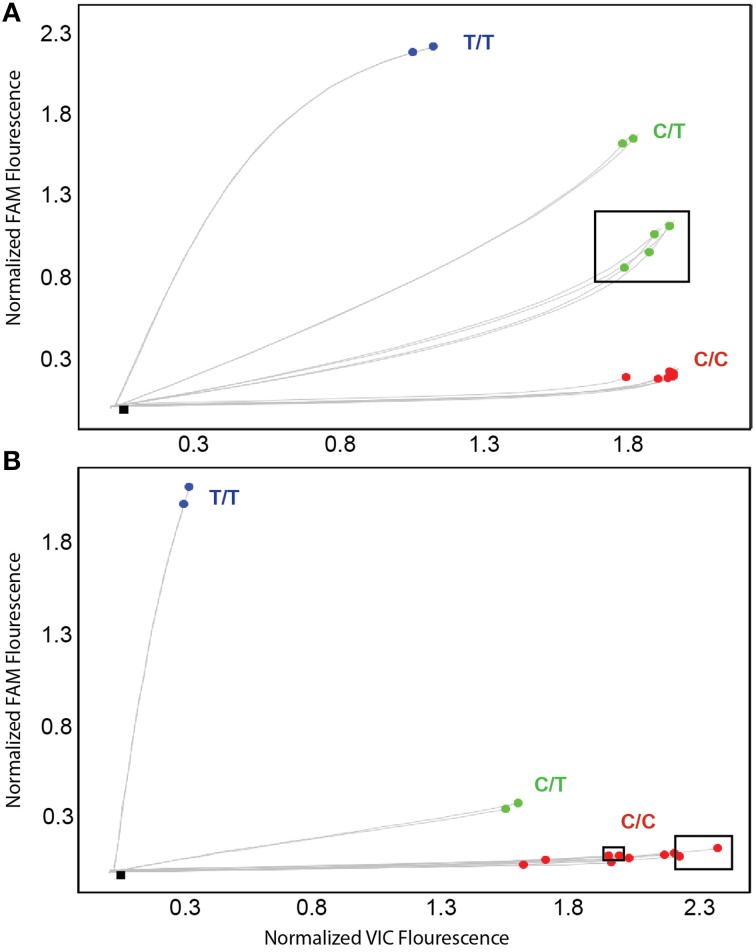
TaqMan amplification profiles generated with the initial (A) and alternate (B) CYP2D6^*^35 TaqMan genotyping assay for false-positive and true positive samples. Profiles shown were generated on WGA-DNA. Boxed samples are false-positives **(A)** that have been resolved with the alternate TaqMan genotyping assay **(B)**.

To address these false-positive calls, alternate CYP2D6^*^15 and ^*^35 assays were designed and evaluated on *CYP2D6* and *CYP2D6* XL-PCR templates, gDNA and WGA-DNA. Figure 3A shows a cluster plot of selected samples for the initial CYP2D6^*^15 assay. As can be seen, the samples identified as false-positives, although on a slightly different trajectory, cluster with true heterozygotes. Since CYP2D6^*^15 is an extremely rare allele, there are typically no, or perhaps single, positive samples in a run besides a heterozygous control sample, making it impossible to distinguish true from false-positives. As shown in Figure 3B, the alternate assay was specific, i.e., the samples that were initially false-positive (shown boxed in Figure 3A) now clustered with wild-type control samples. Furthermore, the alternate assay did not produce any fluorescent signals when *CYP2D7* XL-PCR was used as template, indicating amplification was not supported from *CYP2D7*, including the variants that carry CC>GT (data not shown). Similar results were obtained for the initial and alternate CYP2D6^*^35 assays, respectively (Figure 4). The false-positive samples, however, formed a more distinct cluster when run with the initial assay due to extra VIC signal contributed by amplification of the *CYP2D6* allele. Since CYP2D6^*^35 is a more common allele, false-positives may be easier to provisionally identify compared to CYP2D6^*^15 false-positives; albeit in other experiments run with gDNAs this pattern has been less obvious (data not shown). When run with the alternate assay, the false-positive samples cluster unequivocally with the wild-type controls. Genotyping and sequencing results for all 70 samples as well as NA17128 and two control samples are summarized in Table 1 and Suppl Table 1.

Sequencing of the *CYP2D6* gene in NA17128 revealed SNPs at 77G>A (rs28371696; heterozygous), 310G>T (rs28371699, homozygous), 843T>G, (heterozygous), and 1067T>G (heterozygous), which is consistent with a CYP2D6^*^1var2/^*^43 genotype (^*^1var2 is a ^*^1 subvariant we have recently observed in a trio and described elsewhere (Twist et al., in press). Subsequent TaqMan genotyping of our study cohort revealed that 77G>A was present in 14 samples (Table 1 and Suppl Table 1 and Suppl Figure 1). XL-PCR sequence data also revealed the presence of 77G>A (CYP2D6^*^43) in most subjects initially thought to have a CYP2D6^*^1 allele.

Three of the four sequencing-confirmed samples were also correctly identified with the alternative CYP2D6^*^15 assay, while all false-negative samples returned wild-type calls. Sample #13, however, which was initially genotyped as CYP2D6^*^4/^*^15 (Table 1 and Suppl Table 1) and confirmed by Luminex xTAG genotyping, was not identified by the alternate CYP2D6^*^15 TaqMan assay to have the 137Tins. In addition to the CYP2D6^*^15 137Tins XL-PCR sequencing of the exon 1 region also revealed heterozygosity for 77G>A, a SNP that is part of the CYP2D6^*^43 allele definition, and two novel exon 1 SNPs, 102A>G and 108C>T. As shown in Suppl Figure 1, 102A>G and 108C>T interfere with one of the alternate CYP2D6^*^15 TaqMan assay primers suggesting that these SNPs as well as 77G>A are on the same chromosome as 137Tins forming a novel CYP2D6^*^15var haplotype.

We did not find any evidence that the use of WGA-DNA impacted assay results. Specifically, the redesigned CYP2D6^*^15 and ^*^35 assays were performed on a subset of samples on both gDNA and WGA-DNA. Furthermore, there were no inconsistent findings between WGA-DNA-derived TaqMan assay results and any other results obtained on gDNA including genotype analysis on the Luminex platform or genotyping or sequencing of XL-PCR products that would have indicated erroneous results using WGA-DNA.

Lastly, sequence analysis of the *CYP2D7* gene of NA17128 and comparison to four unique *CYP2D7* sequences (Suppl Table 2) identified CC>GT SNV at positions −1 and −2 (in relation to the ATG start codon), heterozygosity for seven SNPs that were not observed in any of the other *CYP2D7* sequences, as well as a number of SNPs that are present in one or more of the *CYP2D7* sequences it was compared to.

## Discussion

TaqMan assays are carefully designed and evaluated before commercial marketing. However, rare or unknown SNPs located underneath primer and/or probe regions can impact the performance of many genotype assays, including TaqMan (Gaedigk et al., 2015a,b). We have previously also shown that a small cluster of SNPs found within the PCR amplicon, and not under the primer/probe regions, can negatively impact TaqMan genotype assay performance in certain instances (Gaedigk et al., 2015a).

Here we report for the first time that a SNP in the highly similar *CYP2D7* pseudogene interferes with a *CYP2D6* TaqMan genotype assay. In the cases presented, the underlying cause of the false-positive results did not reside within the interrogated gene, but it's near identical *CYP2D7* pseudogene. The high degree of sequence identity between *CYP2D6* and *CYP2D7* makes it extremely difficult to find regions that are gene-specific that fulfill other TaqMan assay requirements including amplicon size. The region immediately upstream of the ATG start codon differs between the two genes by two consecutive nucleotides when available genomic sequences are compared (*CYP2D6* has GT and *CYP2D7* has CC) making this region a good target for primer design. A rare dinucleotide SNP on *CYP2D7*, however, converted the sequence to *CYP2D6*, allowing primer binding as outlined in Figure 1. Consequently, a PCR product was formed from the intended *CYP2D6* target and from the variant *CYP2D7* allele, generating pseudo-heterozygosity. *S*ince *CYP2D7* carries an additional T in exon 1 (corresponding to the CYP2D6^*^15 variant sequence), a mutation signal will be generated by the TaqMan fluorogenic probe off the *CYP2D7* amplification product; as the fluorescent signal is produced from two *CYP2D6* alleles and one *CYP2D7* allele, such samples possess a slightly different trajectory and position in the cluster plot (Figure 3A for CYP2D6^*^15 and Figure 4A for CYP2D6^*^35). Similar observations are made when samples carry a gene duplication, e.g., CYP2D6^*^1/^*^4x2, in which signals are also derived from three loci.

Currently, there are >2000 variant annotations for the *CYP2D7* pseudogene, many with unknown frequencies or minor allele frequencies (MAF) < 0.1% (ENSEMBL, accessed 8-8-2015). Among those is the CC>GT variation which is inventoried by dbSNP under rs386821512 with no population or frequency data. However, this dinucleotide variant appears to be represented in the NCBI dbSNP database as two recently characterized SNPs: a C>G SNP at position −1 (rs556798398) and a C>T SNP at position −2 (rs538594407). These SNPs were identified in the 1000 Genomes (1000 g) project (Abecasis et al., 2012) at an overall population frequency of 0.42% each; a review of the 1000 g phase 3, May 2013 data indicated that both SNPs are found in the same 21 individuals. Similarly, all five samples that were sequenced in our study contained *CYP2D7* CC>GT and HRM patterns of the remaining samples were consistent with the samples carrying CC>GT.

The numerous *CYP2D7* sequence variations annotated in GenBank and ENSEMBL make it almost impossible to negotiate *CYP2D7* variation when designing *CYP2D6* genotyping assays. Moreover, gDNA samples carrying rare alleles are often not available to validate assay performance, i.e., validation that the assay not only detects the allele of interest in heterozygous and homozygous samples, but that no other variant(s) impact the assay's performance. It is therefore prudent to confirm the presence of rare SNPs with a reference method to validate assay performance and avoid reporting false-positive results. TaqMan genotyping assays are extensively tested before being commercially available; however, in situations such as the one described here and reported previously (Gaedigk et al., 2015a,b), rare SNPs, or SNPs in certain genotype constellations, can cause false results that may potentially lead to incorrect phenotype prediction and impact clinical decision making for a patient.

The present work is also the first report establishing the presence of CYP2D6^*^15 on a large population cohort. Although this allele was among the first described and designated in *CYP2D6*, there are sparse data regarding its population distribution and frequency (http://www.pharmgkb.org/download.action?filename=CYP2D6_Frequency_Table_and_Legend_R3.pdf). In our diverse U.S. population, we identified only 15 CYP2D6^*^15 carriers, which corresponds to a prevalence of 0.1% and an allele frequency of 0.05%. Among those, 11 subjects self-identified as Caucasians, one as Asian and three were of unknown ethnicity.

Among the 55 false-positive samples which were confirmed by Luminex genotyping and XL-PCR sequencing, the majority were African American (*n* = 34), followed by Caucasians (*n* = 8), Hispanics (*n* = 7) and those with unknown ethnicity (*n* = 6); NA17128 is also Caucasian. Although there are no frequency data for rs386821512 (CC>GT) per the dbSNP database (accessed 9-30-2015), the MAF data for the apparently linked rs556798398 (C>G) and rs538594407 (C>T) SNPs indicate that they are predominantly found in subjects with African and European ancestry (1 and 0.1%, respectively), which is consistent with the ethnicity information of the subjects identified to carry CC>GT (Table 1 and Suppl Table 1).

As demonstrated, rs386821512 did not only cause false-positive results for CYP2D6^*^15, but also for CYP2D6^*^35. Miscalls for this allele were initially not detected in the cohort because the latter was not part of the custom OpenArray panel employed for genotyping. False-positive calls for CYP2D6^*^35, however, are less consequential because this allele is currently assigned the same activity score value for phenotype prediction as CYP2D6^*^1 (Gaedigk et al., 2008; Hicks et al., 2015). A false-positive result for 31G>A (rs769258) may also be easier to detect than CYP2D6^*^15 false-positives, if 2850C>T (rs16947) and 4180G>C (rs1135840) are part of the test panel. Based on allele definitions, 2850C>T and 4180G>C are expected to be present if 31G>A is detected due to complete linkage of these three SNPs in CYP2D6^*^35. This was indeed the case for NA17128, which was negative for 2850C>T and 4180G>C, as well as for −1584G (rs1080985), which is also part of the CYP2D6^*^35 haplotype and may aid in the identification of false-positives.

After identifying the underlying cause of the false-positive calls, both TaqMan genotype assays were carefully redesigned and validated on our sample set. Three of the four sequencing-confirmed, positives for CYP2D6^*^15 samples were correctly identified, while all false-negative samples returned wild-type calls. The redesigned assays were also tested on *CYP2D7* XL-PCR template to demonstrate that indeed no signal is produced from the *CYP2D7* GT variant. One sample carrying the 137 T-insertion, however, failed to be detected by the alternative CYP2D6^*^15 TaqMan assay due to the presence of two novel SNPs within exon 1, which were only discovered during the course of our investigation. Due to the high sequence similarities between *CYP2D6* and *CYP2D7*, it appears impossible to steer clear of all variation when designing genotyping assays. Reliable detection of CYP2D6^*^15 can tentatively only be achieved by a combination of tests.

Interestingly, we also revealed linkage between the *CYP2D7* GT variant and CYP2D6^*^43. All samples interrogated for and shown to carry CC>GT, were initially false-positive for CYP2D6^*^15, and were subsequently heterozygous for 77G>A (Suppl Table 1). Limited data for CYP2D6^*^43 suggest an allele frequency of about 0.5% (http://www.pharmgkb.org/download.action?filename=CYP2D6_Frequency_Table_and_Legend_R3.pdf), which is similar to that reported for rs538594407 and found in our study cohort (*n* = 55 including subject 13) which corresponds to an overall allele frequency of 0.2%, further supporting linkage between *CYP2D7* CC>GT and CYP2D6^*^43. Six samples that were initially false-positive for CYP2D6^*^15 and also had a CYP2D6^*^1 allele, did not have 77G>A. Because, these samples were not interrogated for *CYP2D7* CC>GT, however, we can only speculate whether *CYP2D7* CC>GT and 77G>A are in complete linkage.

One subject (#13, of unknown ethnicity) was tentatively found to have a novel CYP2D6^*^15 subvariant that is characterized by the presence of two additional *CYP2D6* SNPs, 102A>G and 108C>T, both of which correspond to the *CYP2D7* reference sequence (Supplemental Figure 1). Although we were not able to unequivocally map the two novel SNPs onto the CYP2D6^*^15 allele, we infer this location because these SNPs appear to prevent primer binding of the alternate CYP2D6^*^15 assay, causing allele-drop out. Hence, the assay only detects the wild-type allele on the other chromosome. We have only identified a single subject among 13,866, making this allele extremely rare at (0.004%).

In summary, the discovery and systematic characterization of the underpinning cause of false-positive TaqMan genotyping assay calls led to the development of alternative CYP2D6^*^15 and ^*^35 assays to accurately detect these alleles with one caveat, i.e., a novel, but rare, CYP2D6^*^15 subvariant eludes detection with the alternate assay. Given the sequence similarities between *CYP2D6* and *CYP2D7*, and polymorphisms each gene harbors in the region of interest that mimic each other, it is nearly impossible to design genotyping assays that are *CYP2D6*-specific, steer clear of the *CYP2D7* CC>GT variation and detect all CYP2D6^*^15 including the novel subvariant. One solution may be to perform both the initial and alternate assays to identify true CYP2D6^*^15 alleles. In addition, this study also adds valuable information regarding the frequency of CYP2D6^*^15, produced a complete gene sequence of CYP2D6^*^43, revealed linkage between CYP2D6^*^43 and *CYP2D7* CC>GT and, lastly, but not least important, demonstrates that the presence and variability of a highly related pseudogene complicates genetic testing of *CYP2D6* and can interfere and cause erroneous results.

## Author contributions

AG, MD, and TH designed the study. AR, TH, and KF performed experiments and AG, AR, TH, and MD contributed to data analysis. TH designed alternate TaqMan assays. All authors contributed to the writing of the manuscript.

### Conflict of interest statement

Andrea Gaedigk, Amanda K. Riffel, J. Steven Leeder do not report any conflicts of interest. Mehdi Dehghani, Kristen C. Floyd, Kevin P. Rosenblatt are employees of CompanionDx®; Reference Lab. One of the authors (Toinette Hartshorne) is employed by Thermo Fisher. Assays utilized and evaluated are manufactured and sold by Thermo Fisher Scientific. The alternate assays were made available to participants in this study at no cost.
