# Supplementary material for: CYP2D7 Sequence Variation Interferes with TaqMan CYP2D6*15 and *35 Genotyping
Source: Front Pharmacol. 2016 Jan 12;6:312. doi: 10.3389/fphar.2015.00312 (PMC4709848; doi:10.3389/fphar.2015.00312)
Supplement: Supplementary file 1 [file Table1.PDF]

Suppl Table 1

Summary of all 70 samples initially detected as *CYP2D6*15*-positive

ID current study	Ethnicity	OpenArray Genotype[b]	Luminex Genotype	XL-PCR seq 137T-ins (*15)	XL-PCR seq 77G>A (*43)
1	Unknown	*2A/*15	no call	het	wt
2	African American	*1/*17	*1/*17	wt	het
3	Unknown	*1/*2B	*1/*2B	wt	het
4	Caucasian	*1/*4 (CNV=3)	*1/*4, DUP	wt	het
5	Hispanic	*1/*1	*1/*1	wt	het
6	Unknown	*1/*2D	*1/*29	wt	het
7	Caucasian	*1/*2B (CNV=3)	*1/*2, DUP	wt	het
8	African American	*1/*17	*1/*17	wt	het
9	Hispanic	*1/*2B (CNV=3)	*1/*2, DUP	wt	het
10	African American	*1/*17	*1/*17	wt	het
11	Caucasian	*2A/*15	no call	het	wt
12	African American	*1/*17 (CNV=3)	*1/*17, DUP	wt	het
13	Caucasian	*4/*15	*4/*15	het	het
14	Caucasian	*15/*41	*15/*41	het	wt
15	African American	*2A/*17	*2/*17	wt	wt
16	Hispanic	*1/*17	*1/*17	wt	het
17	African American	*1/*1	*1/*1	wt	het
18	Caucasian	*1/*1	*1/*1	wt	het
19	African American	*1/*41	*1/*41	wt	het
20	African American	*1/*17	*1/*17	wt	het
--	African American	*1/*1	*1/*1	wt	het
--	African American	*1/*1	*1/*1	wt	het
--	African American	*1/*1	*1/*1	wt	het
--	African American	*1/*1	*1/*1	wt	het
--	African American	*1/*10	*1/*10	wt	het
--	African American	*1/*17	*1/*17	wt	het
--	African American	*1/*17	*1/*17	wt	het
--	African American	*1/*2A	*1/*2	wt	wt
--	African American	*1/*2B	*1/*2	wt	het
--	African American	*1/*2B	*1/*2	wt	het
--	African American	*1/*2D	*1/*29	wt	het
--	African American	*1/*2D	*1/*29	wt	het
--	African American	*1/*4	*1/*4	wt	het
--	African American	*1/*4	*1/*4	wt	het
--	African American	*1/*4 (CNV=3)	*1/*4, DUP	wt	het
--	African American	*1/*4 (CNV=3)	*1/*4, DUP	wt	wt

ID current study	Ethnicity	OpenArray Genotype[b]	Luminex Genotype	XL-PCR seq 137T-ins (*15)	XL-PCR seq 77G>A (*43)
--	African American	*1/*5	*1/*5	wt	mut[a]
--	African American	*1/*5	*1/*5	wt	mut[a]
--	African American	*1/*1	*1/1	wt	het
--	African American	*2A/*17	*2/*17	wt	wt
--	African American	*2B/*2B	*2/*2	wt	wt
--	African American	*2A/*4	*2/*4	wt	wt
--	African American	*2A/*5	*2/*5	wt	wt
--	African American	*2A/*5	*2/*5	wt	wt
--	African American	*2B/*4 (CNV=3)	*4/*4, DUP	wt	wt
--	African American	*9/*17	*9/*17	wt	wt
--	Asian	*2/*15	*2/*15	het	wt
--	Caucasian	*1/*15	*1/*15	het	wt
--	Caucasian	*1/*15	*1/*15	--	--
--	Caucasian	*1/*15	*1/*15	het	wt
--	Caucasian	*1/*2A	*1/*2	wt	het
--	Caucasian	*1/*2A (CNV=3)	*1/*2, DUP	wt	wt
--	Caucasian	*1/*6	*1/*6	wt	het
--	Caucasian	*2A/*15	*2/*15	--	--
--	Caucasian	*2/*15	*2/*15	het	wt
--	Caucasian	*2A/*15	*2/*15	het	wt
--	Caucasian	*2A/*4	*2/*4	wt	wt
--	Caucasian	*4/*41	*4/*41	wt	wt
--	Caucasian	*9/*15	*9/*15	--	--
--	Caucasian	*3/*15 or *6/*15	no call	--	--
--	Hispanic	*1/*1	*1/*1	wt	het
--	Hispanic	*1/*1	*1/*1	wt	het
--	Hispanic	*1/*2A	*1/*2	wt	het
--	Hispanic	*1/*2D	*1/*29	wt	het
--	Unknown	*1/*1	*1/*1	wt	het
--	Unknown	*1/*10 + *36	*1/*10	wt	het
--	Unknown	*1/*15	*1/*15	het	wt
--	Unknown	*15/*35	*15/*35	het	wt
--	Unknown	*2A/*10 (CNV=4)	*2/*10,DUP	wt	wt
--	Unknown	*4/*4	*4/*4	wt	wt

Samples confirmed positive for *CYP2D6*15* are highlighted in grey.
All samples with a red "***1***" were positive for 77G>A suggesting the presence of a *CYP2D6*43*.
[a] Sample genotypes as homoygous mutant due to the presence of the *CYP2D6**5 gene deletion allele
CYP2D6-specific XL-PCR template could not be obtained for four samples as indicated by --.

[b] OpenArray genotyping results were obtained with the original *CYP2D6*1*5 TaqMan assay.
