# Supplementary material for: CYP2D7 Sequence Variation Interferes with TaqMan CYP2D6*15 and *35 Genotyping
Source: Front Pharmacol. 2016 Jan 12;6:312. doi: 10.3389/fphar.2015.00312 (PMC4709848; doi:10.3389/fphar.2015.00312)
Supplement: Supplementary file 2 [file Table2.PDF]

Suppl Table 2

Comparison of *CYP2D7* sequences.

Reference		ENSG-205702	ENSG-278088	ENSG-263181	M33387	NA17128	Total
-217	G				C		1
-203	T				C		1
-189	T				C		1
-185.3	:				C		1
-185.2	:				A		1
-185.1	:				C		1
-155	C				G		1
-154	A				G		1
-86	C		T		T	T	3
-2	C					S	1
-1	C					Y	1
311	G				:		1
731	C					Y	1
746	C		G	G	G	G	4
843	T		G		G	G	3
974	A					M	1
1,047	G					S	1
1,234	G					S	1
1,319	C				:		1
1,569	C		T		T	T	3
1,590	A		T		T		2
1,596	T				G		1
1,762	A		C		C		2
1,797	A		G		G	G	3
1,829	G				C		1
1,830	C				G		1
1,848	C		T		T		2
1,996	G		A		A	A	3
1,998	G		:		:	:	3
1,999	A		:		:	:	3
2,000	G		:		:	:	3
2,001	A		:		:	:	3
2,002	C		:		:	:	3
2,003	C		:		:	:	3
2,004	G		:		:	:	3
2,005	A		:		:	:	3
2,006	G		:		:	:	3
2,008	A		G		G	G	3
2,013	C		T		T	T	3
2,076	A		G		G		2
2,246	T		C		C		2
2,372	G		A		A		2

2,446	C		T		T	T	3
2,555	A				C		1
2,586	A				G		1
2,590	A				T		1
2,642	C					Y	1
2,754	G					R	1
2,878	C		T		T		2
3,071	G		C		C		2
3,204	G		A		A	A	3
3,234	A		G	G	G	G	4
3,248	G		A	A	A	R	4
3,257	G		A	A	A	R	4
3,279	G		A		A		2
3,348	C		T		T		2
3,356	T		:		:		2
3,361	A		G		G		2
3,453	A				G	R	2
3,502	C			A			1
4,005	A					R	1
Total		0	34	5	51	32	122

We identified six *CYP2D7* sequences in GenBank and ENSEMBL of which four unique sequences are shown (ENS00000612115 and NC_000022.11 of GRCh38.p2 correspond to ENS00000205702 and are not shown). The two haplotypes composing the *CYP2D7* alleles of NA17128 do not match any of the reference sequences. +1 denotes the ATG start codon. M33387 denotes a GenBank entry; the ENSEMBL identifiers for ENSG-205702, ENSG-205702, ENSG-278088 and ENSG-263181 are ENSG00000205702, ENSG00000205702, ENSG00000278088 and ENSG00000263181, respectively. ENSG-205702 served as reference sequence for the variation report.
