# Supplementary material for: CYP2D7 Sequence Variation Interferes with TaqMan CYP2D6*15 and *35 Genotyping
Source: Front Pharmacol. 2016 Jan 12;6:312. doi: 10.3389/fphar.2015.00312 (PMC4709848; doi:10.3389/fphar.2015.00312)

**Suppl Figure 1**

Graphic display of the *CYP2D6* and *CYP2D7* exon 1 regions of NA 17128 (A) and sample #13 (B). Panel (A) Most samples that were initially false-positive for *CYP2D6*15* that have the variant *CYP2D7* CC>GT allele, including NA17128, carried an allele that defaulted to a *CYP2D6*1* assignment (Table 1 and Suppl Table 1). Sequencing revealed the presence of 77G>A in all but six of those samples, revising the *CYP2D6*1* allele assignment to *CYP2D6*43*. In these six samples, *CYP2D7* CC>GT variation was not determined. (B) Sample #13 does not carry the *CYP2D7* CC>GT variant and is accurately identified by the original *CYP2D6*15* assay as heterozygous for the 137T insertion (Table 1 and Suppl Table 1). However, three additional SNPs, 77G>A and two novel SNPs, 102A>G and 108C>T were revealed by sequencing. The alternate *CYP2D6*15* assay fails, however, to detect the 137T insertion which is likely due to the presence of 102A>G and 108C>T which interfere with primer binding. The approximate primer binding location is shown; an "X" indicates that a primer does not generate PCR product.

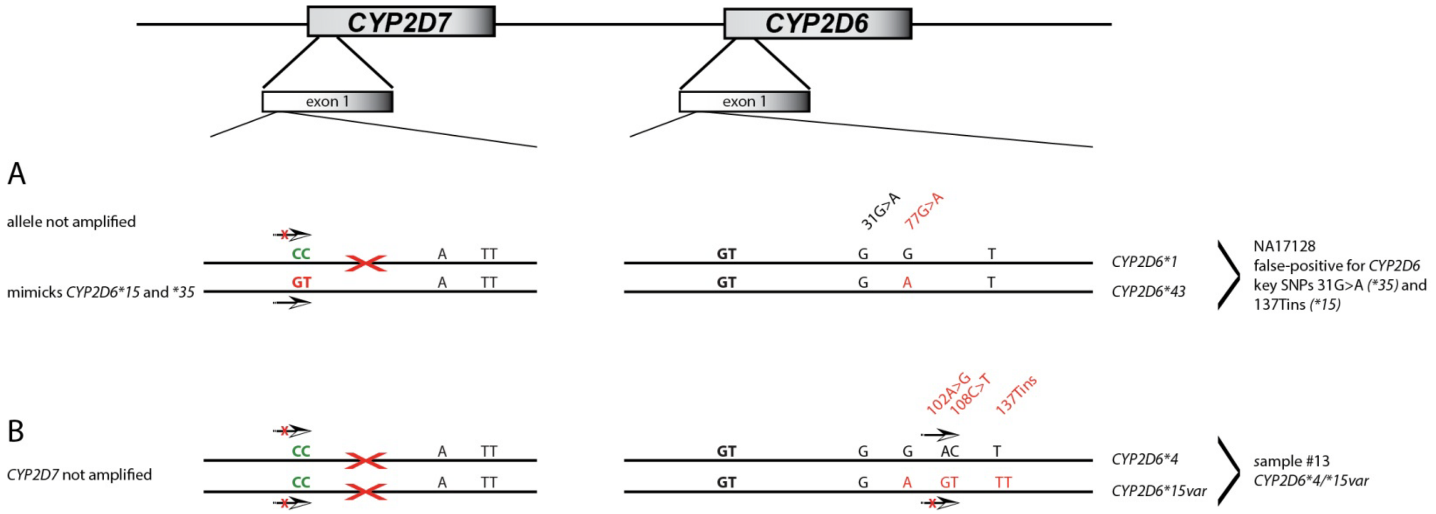

Supplement: Supplementary file 3 [file Image1.PDF]
